# Control of LTR transcription by SWI/SNF BAF and PBAF complexes

**DOI:** 10.1186/1742-4690-11-S1-O47

**Published:** 2014-01-07

**Authors:** Irene Guendel, Rachel Van Duyne, Fatah Kashanchi

**Affiliations:** 1 School of Systems Biology, National Center for Biodefense & Infectious Diseases, George Mason University, Manassas, Virginia, USA; 2 Department of Microbiology, Immunology, & Tropical Medicine, The George Washington University Medical Center, Washington, D.C., USA

## 

The transcription of HTLV-1 LTR is a highly regulated process by the organized chromatin structure within a cell's nucleus; chromatin is folded into compact fibers (30-400 nm thick), which physically prevents the accessibility of cellular promoters by transcriptional machinery. A variety of enzymes and protein complexes are present in the cell to mediate the opening” and “closing” of chromatin structure to allow for viral and cellular processes to occur. This chromatin remodeling occurs through two major mechanisms: covalent epigenetic modifications of N-terminal histone tails and ATP-dependent chromatin remodeling complexes (CRCs). HTLV-1 Tax has been shown by us and others to effectively regulate both steps. Here, we will discuss the effect of BAF and PBAF using a series of siRNA experiments for both basal and activated transcription. We observed that BAF complexes are regulated by phosphorylation of Baf 53 subunit (possibly by pTEF-b) and PBAF complexes substitute the negative inhibitory BAF complexes needed for activated transcription. We believe that Baf 53 activity is through closing of the chromatin structure at the transcription start site and that PBAF is able to aid in elongation by communicating with p300/PCAF and histone acetylated tails. Implications of these complexes in transcription for both viral and cellular genes will be discussed.

